# Stigma associated with genetic testing for rare diseases—causes and recommendations

**DOI:** 10.3389/fgene.2024.1335768

**Published:** 2024-04-04

**Authors:** Gareth Baynam, Roy Gomez, Ritu Jain

**Affiliations:** ^1^ Undiagnosed Diseases Program -WA, Genetic Services of WA, King Edward Memorial Hospital, Subiaco, WA, Australia; ^2^ Western Australian Register of Developmental Anomalies, King Edward Memorial Hospital, Subiaco, WA, Australia; ^3^ Rare Care Centre, Perth Children’s Hospital, Subiaco, WA, Australia; ^4^ Emerging Asia Medical Lead–Specialty Care, Pfizer, Singapore, Singapore; ^5^ Nanyang Technological University, Singapore, Singapore; ^6^ DEBRA International, Asia Pacific Alliance of Rare Disease Organizations, Singapore, Singapore

**Keywords:** stigma, rare diseases, genetic testing, genetic screening, orphan diseases, diagnosis

## Abstract

Rare disease (RD) is a term used to describe numerous, heterogeneous diseases that are geographically disparate. Approximately 400 million people worldwide live with an RD equating to roughly 1 in 10 people, with 71.9% of RDs having a genetic origin. RDs present a distinctive set of challenges to people living with rare diseases (PLWRDs), their families, healthcare professionals (HCPs), healthcare system, and societies at large. The possibility of inheriting a genetic disease has a substantial social and psychological impact on affected families. In addition to other concerns, PLWRDs and their families may feel stigmatized, experience guilt, feel blamed, and stress about passing the disease to future generations. Stigma can affect all stages of the journey of PLWRDs and their families, from pre-diagnosis to treatment access, care and support, and compliance. It adversely impacts the quality of life of RD patients. To better explore the impact of stigma associated with genetic testing for RDs, we conducted a literature search on PubMed and Embase databases to identify articles published on stigma and RDs from January 2013 to February 2023. There is a dearth of literature investigating the dynamics of stigma and RD genetic testing. The authors observed that the research into the implications of stigma for patient outcomes in low- and middle-income countries (LMICs) and potential interventions is limited. Herein, the authors present a review of published literature on stigma with a focus on RD genetic testing, the associated challenges, and possible ways to address these.

## 1 Introduction

Rare disease (RD) is a term used to describe numerous, heterogeneous diseases that are geographically disparate. There is no universal definition for RDs, and their understanding varies depending on the political and legislative framework of each country (Nguengang et al., 2020). Approximately 400 million people worldwide live with an RD (1) equating to roughly 1 in 10 people which is comparable to that of type II diabetes (Walewski et al., 2019; World Health Organization, 2019). Approximately 10,000 known RDs have been identified (The Global Genes, 2023; RARE-X, 2022; U.S. Department of Health and Human Services, 2023). According to the Orphanet database, 71.9% of RDs have genetic etiology (Nguengang et al., 2020). Many RDs cause severe disability and significantly limit life expectancy and significantly contribute to mortality in impacted children, at least in high-income countries (Makarova et al., 2021).

RDs present a distinctive set of challenges to patients, their families, healthcare professionals (HCPs), healthcare system, and societies at large. There are a number of challenges for people living with rare diseases (PLWRDs), which include a) delay in detection and/or diagnosis of the disease; b) underdeveloped patient communities or patient advocacy groups for individual RDs; c) difficulties in recruitment for clinical trials required for development and registration of potentially promising treatments; and d) access or availability to promising new treatment modalities such as gene therapies (IFPMA, 2023). Some of the reasons for these challenges are lack of sufficient knowledge of the disease, inadequate RD diagnostic infrastructure, lack of or outdated policies, and lack of or insufficient funding (IFPMA, 2023).

RDs are often characterized as “diagnostic odyssey,” with the average time to arrive at a correct RD diagnosis after presentation being 5 years. The diagnostic delays can impede treatment initiation and causes substantial psychological, emotional, and financial distress for patients and their caregivers (Dwyer et al., 2022). Clinical genetic testing helps identify DNA anomalies that cause rare genetic disease. The objective of testing is to diagnose or “predict the risk of developing disease and transmitting disease-causing variants to offspring” (Zhong et al., 2021). Additionally, genetic counseling helps patients understand the test results and their consequences. There are limited genetic testing services available in low- and middle-income countries (LMICs), and they are often provided through research initiatives or formal international partnerships rather than being functionally embedded in healthcare systems; and genetic counseling is yet to evolve to meet the requirements (Zhong et al., 2021).

RDs pose substantial challenges to PLWRDs and their families, as well as to the clinicians who care for them. Patients suffering from RD may struggle with finding an appropriate and knowledgeable physician who can diagnose and manage their condition. The challenges faced by clinicians include limited knowledge and/or experience with RDs. Hence, reasonably, a clinician’s expertise in managing a disease is proportional to the frequency with which they encounter and manage patients with the disease (Stoller, 2018) and the education and training received by them. Additional challenges for both patients and physicians are access and/or availability of the therapy (Ferreira, 2019) and its cost (Stoller, 2018). Securing a diagnosis of an RD impacts patients of all ages at multiple levels. The impact encompasses social, personal, and medical consequences (Esquivel-Sada and Nguyen, 2018). Delayed diagnosis, misdiagnosis, and/or lack of therapies are common challenges faced by PLWRDs (Zanello et al., 2022). Identifying a precise genetic diagnosis can improve outcomes for PLWRD (Zanello et al., 2022; Wojcik et al., 2023). Additionally, the possibility of inheriting a genetic disease has a substantial social and psychological impact on the affected families. Among other concerns, the families and PLWRD may feel stigmatized, experience guilt, blame parent(s), or even worry about passing the disease to future generations. Collectively, these hamper the ability of PLWRDs as well as their families to adjust to the disease (James et al., 2006).

Stigma can be described as ‘‘an attribute that is deeply discrediting’’ or as a ‘‘mark’’ or “aspect of the self that is socially devalued.” Stigma may be a result of the ‘‘mark’’ itself or of social interactions during which ‘‘mark’’ is perceived as a reflection of its possessor’s tainted characteristic (Earnshaw and Chaudoir, 2009). Perceived stigma refers to “a person’s understanding of how others may act toward, and think or feel about, an individual with a certain trait or identity” (Zelaya et al., 2012). Anticipated stigma refers to “expectations of stigma experiences happening in the future” (Earnshaw et al., 2013). Internalized stigma refers to “the individual level process of awareness, acceptance, and application of stigma” (Munoz et al., 2011). Experienced or enacted stigma refers to “discriminatory acts or behaviors” (Catona et al., 2016). Stigma may result in poor health outcomes, due to its adverse impact on help- and treatment-seeking behaviors, such as searching for a definitive diagnosis, among patients across a range of diseases (Kane et al., 2019).

Despite the rich work on stigma in other disease domains, e.g., HIV (Tan et al., 2020), studies examining the impact of stigma among PLWRDs remain sparse, especially in children and in LMICs. The National Institute of Mental Health (NIMH) in partnership with the Fogarty International Center (FIC), the National Institute on Drug Abuse (NIDA), and the National Institute of Health (NIH) Stigma Scientific Interest Group has developed the Stigma and Discrimination Research toolkit. This toolkit is helpful for researchers, government officials, community agencies, and other relevant stakeholders (National Institute of Mental Health, 2024). The Health Stigma and Discrimination Framework published by Stangl et al. (2019) contextualizes stigma across the socioecological spectrum that differs across low-, middle-, and high-income countries. The framework provides a process divided in sub-domains, which includes drivers and facilitators, stigma “marking”, and stigma manifestations. Stigma manifestation influences outcomes among affected populations and organizations/institutions, which ultimately impact health and society (Stangl et al., 2019). One of the chief benefits of implementing a framework to understand stigma is the provision to recognize health-related stigma as a co-occurrence with other intersecting stigmas. The intersecting stigmas include sexual orientation, gender, race, occupation, and economic conditions. To understand the full impact of stigma on health outcomes, including intersecting stigmas into the framework is crucial (Stangl et al., 2019). Although the framework can be used for communicable and non-communicable diseases, all domains are not applicable across all heath conditions despite some level of commonality. There are no specific frameworks emphasizing on stigma associated with RDs or genetic diseases. In order to develop a similar framework for RDs considering their nuances, it is first crucial to understand the source and severity of stigma.

Notwithstanding that stigma associated with RDs is understudied, some research studies have found RD stigmatization to be associated with poor quality of life (Bogart et al., 2022). While economic factors clearly influence diagnostic access, they may also simultaneously reduce research into systemic, socially driven barriers (such as stigma) to accessing genetic testing in LMICs. The relative absence of community awareness, engagement in advocacy activities, and connectivity to stakeholders (Chediak et al., 2022) in LMICs may also be factors that have limited investigation of stigma in these countries.

In this review, the authors assess the stigma associated with genetic testing of RDs, the associated challenges, and possible ways to address these.

## 2 Methods

A literature search of PubMed and Embase databases was conducted for articles published from January 2013 to February 2023. The keywords used included Stigma, Genetic testing, Genetic screening, RDs, Perceptions, Psychological impact, Orphan diseases, Counselling, Risk communication, Sociocultural factors, Diagnosis, Equity, Fear, Disease-related stigma, Health-related felt stigma, Genetic discrimination, Disease related stigma scale, Self-stigma measures, Psychometric evaluation of stigma, Quality of life, Shame, and psychological distress. The search strings used were (Stigma) AND ((Genetic testing) OR (Genetic screening)); (Stigma) AND ((Genetic testing) OR (Genetic screening)) AND ((Rare Diseases) OR (Orphan Diseases)) (Stigma) AND ((Genetic testing) OR (Genetic screening)) AND (Diagnosis); (Stigma) AND (Diagnosis) AND ((Rare Diseases) OR (Orphan Diseases)); ((Genetic testing) OR (Genetic screening)) AND (Fear); ((Genetic testing) OR (Genetic screening)) AND (Perceptions); ((Genetic testing) OR (Genetic screening)) AND ((Rare Diseases) OR (Orphan Diseases)) AND (Risk communication); ((Genetic testing) OR (Genetic screening)) AND ((Rare Diseases) OR (Orphan Diseases)) AND (Equity); ((Genetic testing) OR (Genetic screening)) AND ((Rare Diseases) OR (Orphan Diseases)) AND (Sociocultural factors)); ((Genetic testing) OR (Genetic screening)) AND ((Rare Diseases) OR (Orphan Diseases)) AND (Psychological Impact); ((Rare Diseases) OR (Orphan Diseases)) AND (Psychological Impact) AND (Counselling); ((Rare Diseases) OR (Orphan Diseases)) AND (Counselling); ((Rare Diseases) OR (Orphan Diseases)) AND (Disease-related stigma); ((Rare Diseases) OR (Orphan Diseases)) AND (Health-related felt stigma); (Stigma) AND ((Rare Diseases) OR (Orphan Diseases)) AND (Genetic discrimination); ((Rare Diseases) OR (Orphan Diseases)) AND (Disease related stigma scale); ((Genetic testing) OR (Genetic screening)) AND ((Rare Diseases) OR (Orphan Diseases)) AND (Self-stigma measures); ((Genetic testing) OR (Genetic screening)) AND ((Rare Diseases) OR (Orphan Diseases)) AND (Psychometric evaluation of stigma); (Shame) AND ((Genetic testing) OR (Genetic screening)); (Shame) AND ((Genetic testing) OR (Genetic screening)) AND ((Rare Diseases) OR (Orphan Diseases)); (Shame) AND ((Genetic testing) OR (Genetic screening)) AND (Diagnosis); (Shame) AND (Diagnosis) AND ((Rare Diseases) OR (Orphan Diseases)) ((Rare Diseases) OR (Orphan Diseases)) AND (Quality of life); ((Genetic testing) OR (Genetic screening)) AND ((Rare Diseases) OR (Orphan Diseases)) AND (Psychological Distress); and ((Rare Diseases) OR (Orphan Diseases)) AND (Psychological Distress).

The search yielded a total of 13,344 results. The articles were screened using titles and abstracts to remove duplicates and articles not containing relevant information. Original/research articles, reviews, systematic reviews, meta analyses, case reports, letter to the editor, and short communications discussing stigma associated with genetic screening of RDs were included.

The authors conducted an open-label selection of the articles which contained relevant information. Article titles, abstracts, link to full text of the articles, and citations were shared with the authors. The authors assessed the articles and selected the relevant articles for inclusion if they discussed about stigma associated with RDs, the reasons for stigma, type of stigma, and impact of stigma on genetic testing of RDs.

Articles not in English and discussing stigma associated with genetic screening of cancers and other diseases were excluded. Eventually, a total of 44 articles were found to be relevant and subjected to voting by the authors.

**FIGURE 1:**
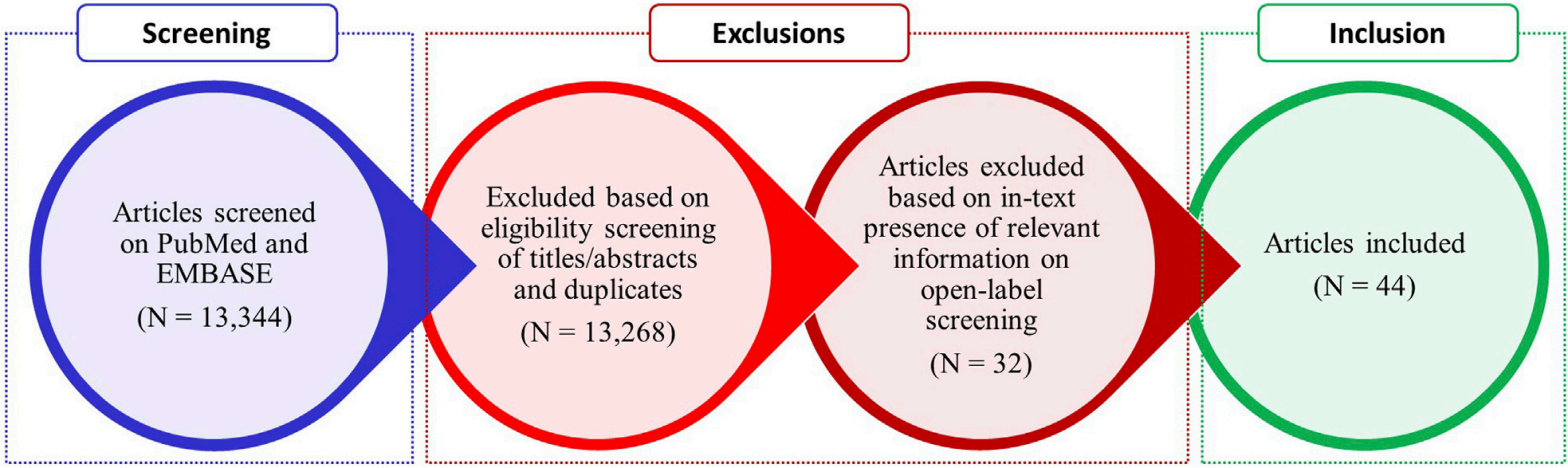
Schematics of literature search.

Figure 1 showcases the results of literature search.

## 3 Barriers to rare disease diagnosis

### 3.1 Diagnostic challenges

PLWRDs face hurdles such as delay in receiving a diagnosis, incorrect diagnosis, and lack of treatment modalities (Zanello et al., 2022). The unmet medical and social needs of patients and families are present globally, despite the efforts undertaken to improve the diagnostic capabilities. Despite technological advances, there are disparities related to obtaining a correct diagnosis and access to care owing to geographic, socioeconomic, and cultural variations within and between countries. Similarly, a combination of cultural, ethical, legal, and social issues is associated with stigmatization related to disease diagnosis (Groft et al., 2021). These concerns can be addressed by providing early access to diagnosis and therapies that can impact management and the progression of diseases, which in return favorably impacts PLWRDs, families, and healthcare systems (Zanello et al., 2022). Achieving a precise diagnosis requires a comprehensive knowledge of the genetic pathogenesis and accessibility to the required diagnostic tools. The foundation of genetic testing or genomic medicine is dependent on complete understanding of an RD genome and on cataloging local genomes, understanding of all causal RD genotypes, and subsequent phenotypes (Boycott and Ardigo, 2018; Chediak et al., 2022). Part of the challenge is realizing when the benchmark of sufficient understanding has been reached as there is far from a 1:1 connection between disease genes and RDs, with >30% of disease genes causing more than one RD secondary to pleiotropy (Boycott and Ardigo, 2018).

### 3.2 Genetic testing and counseling

Genetic counseling helps patients with genetic disorders and their families understand the results and consequences of genetic testing. As technology has evolved and genetic tests are increasingly available, the need for genetic counseling is escalating (Zhong et al., 2021). Accordingly, in a “Patient Attitudes and Beliefs” survey, it was observed that patients with congenital hypogonadotropic hypogonadism (CHH) are driven by altruism to pursue genetic testing. However, there is a substantial unmet need for genetic counseling to support pretest decision making and post-test counseling (Dwyer et al., 2022). Furthermore, a survey evaluating the opinion of the members of RD social media groups on engaging with genetic counselors through social media found that PLWRDs and their family members were interested in connecting with genetic counselors through these platforms (Yabumoto et al., 2022).

With the technological advances in genomics, genetic screening offers numerous benefits: determining a diagnosis, promoting individualized management, providing information on prognosis and recurrence risk, facilitating access to patient support groups, and enabling education, clinical, and social care (Verberne et al., 2022). Genetic testing is crucial to determine precise diagnosis and to devise a suitable treatment approach in many cases. For patients with transthyretin (TTR)-related hereditary amyloidosis, genetic screening is required to determine the pathogenic mutation. Symptoms and stage of disease progression can be further determined using a number of disease-specific criteria including neurologic tests and the modified body mass index. Hence, an accurate diagnosis is crucial to decide the standard of care (Ando et al., 2013). Similarly, The Endocrine Society endorses genetic counseling and testing to patients with multiple endocrine neoplasia type 1 (MEN1) and also to their first-degree relatives to check for inherited endocrinopathies which are rare and are linked with substantial morbidity and mortality (Gallagher et al., 2017).

Genetic testing often raises the concern of “risk of knowing” which has a connotation of being associated with negative psychosocial and interpersonal implications of one’s genetic status (Yau and Zayts, 2014). Research on genetic counseling communication has demonstrated that the “risk of knowing” conversation involves an active part of the counseling agenda, which is usually initiated by HCPs (Yau and Zayts, 2014). Genetic testing is seldomly seen as a “benefits of knowing,” which is in contrast to “risk of knowing,” and emphasizes on the positive implications of knowing about one’s genetic status (Yau and Zayts, 2014).

One of the challenges to use genetic testing is insufficient practical guidance on access and cost/insurance for genetic testing for RDs (Robillard et al., 2021). Another challenge to genetic testing is the availability and accessibility of diagnostic facilities. Numerous patients with a suspected RD can only undergo genetic testing through participation in a research study. Conducting genetic tests for RDs is sometimes unattractive to clinical laboratories owing to their perceived low profitability. Lack of knowledge and grasp of fast-paced developments in the field of genetic testing among HCPs is an additional barrier to accessing genetic testing (Kruse et al., 2021). As a result, improving clarity on publicly available resources on genetic testing is imperative for encouraging the patient community to make informed choices about the procedure, mitigate potential harms associated with lack of information, and enable greater engagement in their own healthcare (Robillard et al., 2021).

Genetic testing is also associated with ethical challenges at an individual, organizational, and macro level of healthcare systems. To conduct a program for genetic testing for RDs, one needs a thorough understanding of the complexity and multiplicity of the ethical concerns. Another obstacle for obtaining a genetic diagnosis is the cost and insurance coverage (Srinivasan et al., 2020).

*Best et al.* in their systematic review analyzed barriers and enablers to receiving genetic services based on the geographical location for non-cancer-related RDs. The barriers included lack of awareness among patients and clinicians, distance to the testing facility, role of cultural and religious beliefs, opportunity costs, bandwidth of rural workforce, logistical issues, lack of required skills, lack of investment, distribution of workforce capable of conducting genetic testing, and paucity of opportunities. The enablers identified for genetic testing were a growth opportunity for geneticists, innovative models of care, educational opportunities, opportunity for building partnerships with geneticists, development of locally relevant implementation strategies, and need assessment (Best et al., 2022).

Prenatal genetic testing that allows screening a fetus for possible genetic disorders is also met with challenges (Zhong et al., 2021). The negative perception about genetic disorders and the perceived lack of medical support/treatment options may influence people to terminate the pregnancy (if legally allowed). The influence of spouse and family members significantly affects the decision regarding how to deal with the results of prenatal genetic testing (Zhong et al., 2021). The study by *Yau et al.* highlights that after prenatal screening, during counseling, participants vocalized concerns about having a child with Down’s syndrome (Yau and Zayts, 2014). Similarly, in another qualitative study, published by *Phipps et al.*, participants undergoing prenatal testing for Muenke disease shared their apprehensions regarding sharing the information about diagnosis with family and friends. The fear of stigmatization was observed to be overwhelming (Phipps and Skirton, 2017).

Although the challenges to genetic testing vary as per the healthcare structure of a particular country, social stigmatization and the apprehensions remain a common theme.

## 4 Stigma associated with genetic testing

Stigma is considered a hidden burden of disease by the World Health Organization (WHO) and is described by cognitive, emotional, and behavioral components. Stigma is reflected in the attitudes of individuals and is conceptualized as perceived, anticipated, or internalized stigmas. It is also reflected in the experiences of individuals, including enacted or experienced stigmas (Kane et al., 2019).

Many PLWRDs experience stigma allied with genetic discrimination, which may occur with behaviors of labeling, stereotyping, separation, and status loss. Stigma creates the perception of negative characteristics about the stigmatized person, which suggests a diminished social identity (Williams et al., 2010). Stigma can affect all stages of the journey of PLWRDs and their families, from pre-diagnosis to access to treatment, care and support, and compliance. It adversely impacts the quality of life of RD patients and their families/caregivers.

Stigma and discrimination are presented in various forms including regulatory issues, insurance or employment, or social issues such as exclusion from social activities (Kruse et al., 2021). Stigmatization is experienced not only by the people with a particular diagnosis but also extended to those with a positive carrier status. This discourages the implementation of cascade screening and population carrier screening (Kruse et al., 2021). Stigma and genetic discrimination are not universal experiences for everyone diagnosed with a genetic disease. However, unaddressed stigma can hamper genetic test access (Kruse et al., 2021). Furthermore, in some cultural contexts, stigmatization could cause gender-based discrimination and reproductive restrictions (Kruse et al., 2021). The fear of discrimination induced by stigma leads to hesitancy in receiving proper information and treatment to manage the condition. This ultimately leads to worse or suboptimal outcomes among PLWRDs.

In a survey of families and people living with Fragile X syndrome, *Boardman et al.* observed that families living with Fragile X syndrome generally support genetic population screening, but with some skepticism. The participants were more accepting of pre-conception genetic screening over prenatal screening. The participants expressed that the heightened stigma associated with cognitive/intellectual disability would be further “underscored and left unchallenged” by changing Fragile X syndrome into a “screened-for” condition (Boardman, 2021). Whole-genome sequencing has also raised concerns about the inappropriate use of genomics data, which may lead to legal or financial complications in addition to stigmatization and employment discrimination (Koromina et al., 2021).

*Boeldt et al.* assessed the perspectives of adult patients and parents of children who were offered diagnostic whole-genome sequencing. The most cited benefit by the participants was the possibility of collecting information or insight on patients’ condition. Participants believed that undergoing genome sequencing could allow medical researchers to identify other diseases or genetic predispositions and better understand the drug interactions for more effective treatment outcomes. The parents and patients were interested in knowing about the genetic condition, provided the information would be useful or actionable. Even though the participants were aware that the process could result in devastating findings or inconclusive results, some were hopeful that the results will help researchers to discover something helpful for others in the future. Participants also expressed an emotional release and gaining closure from new knowledge about the previously unidentified conditions. The perceived drawback of whole-genome sequencing was the risk of receiving inconclusive results or results with no clinical action available. Findings also indicate an absence of preparedness toward lack of available treatment options post-diagnosis. Participants vented frustration about feeling helpless to improve their or their child’s condition (Boeldt et al., 2017). Another study highlighted that women are afraid of bearing children with genetic abnormalities and are reluctant to share their concerns with others. The burden of knowing that the fetus may possibly have a genetic condition was distressing and overwhelming. A cultural connotation and shame were observed to be associated with having a child with genetic condition (Jun et al., 2017). Cultural and religious beliefs contribute to apprehensions and fears of genetic testing. For various population groups, communities, ethnic cultures and religions, social factors, and stigma may play a prominent role in influencing perceptions about a disease severity. This has adverse consequences for affected families. These factors also contribute to the decision associated with termination of pregnancies since there is a substantial fear of being “blacklisted” following the discovery of carrier status and of being “shunned” by family and community members following a pregnancy termination (Boardman et al., 2020). A study conducted by Tsai et al., among Southeast and East Asian women in the United States, discovered that the participants were more likely to weigh risks and benefits with regard to genetic testing decisions and had mixed views on termination for lethal and non-lethal genetic conditions. The cultural factors had an evident influence on attitude toward genetic screening (Tsai et al., 2017).

Table 1 summarizes stigma/perceptions about genetic screening identified in the literature.

**TABLE 1:** Literature summary of stigma/perceptions about genetic screening.

Sr No.	Condition/disease/disorder	Proportion of participants favoring genetic screening	Highlighted stigma
1	Cystic fibrosis (Anton-Paduraru, 2017)	-	• Stress associated with carrier status, especially when only one parent is a carrier
			• Frequent clinic visits from an early age
2	Errors of metabolism (Beck et al., 2020)	-	• Isolation
			• Shame/guilt
			• Grief
			• Anxiety and depression
3	Spinal muscular atrophy (Boardman et al., 2017; Boardman et al., 2018)	75% (n = 337)	• Carrier stigmatization
			• Eugenics/social engineering
4	RDs (Bonneau et al., 2021)	57% (n = 1,568)	• Eugenics
			• Over-medicalization of procreation
			• Undue stress
5	Complex diseases (Fagbemiro and Adebamowo, 2014)	• 100% for themselves (n = 80)	• Data privacy
		• Majority did not agree for *in utero* genomic screening	• Religious beliefs
6	Sickle cell disease (SCD) (Naik and Haywood, 2015; Kisanga et al., 2021)	• NA (Beck et al., 2020)	• Racial discrimination
		• 100% (n = 10 parents of children with SCD) (Boardman et al., 2017)	• Community fear/mistrust
			• Incomplete knowledge
			• Social implications
			• Carrier status
			• Lack of understanding leading to assumption of HIV in SCD patients
7	Familial melanoma (Primiero et al., 2021)	-	• Generalized anxiety
			• Depression
			• Distress/worry/concern
			• Regret

Stigma impacts the quality of life of patients with RDs and their parents/caregivers. Patients often report experiences of structurally enacted stigma wherein patients face invalidation and disbelief by healthcare practitioners, an overall lack of support at the workplace, and social discrimination. People with RDs experience a lack of understanding or recognition from surrounding people and receive insufficient social support. Patients tend to internalize this stigma and feel shame or pressure to hide their condition. Parents also tend to experience frustration due to feelings of isolation and lack of knowledge (Ayres et al., 2019; Bogart et al., 2022). Furthermore, parents of children with RDs fear the long-term progression of the child’s disease and the loss of their parental role. The impact on quality of life and mental health has been observed to be more in mothers than in fathers (Boettcher et al., 2020). Genetic counseling may help in reducing the worry among parents, even if there is limited or no specific management or treatment for their child (Ayres et al., 2019).

Socioeconomic and cultural differences lead to unique challenges and impact on PLWRD and their families. The response to genetic conditions could be governed by cultural belief systems, which result in shame and social stigma with consequences. Some cultures enforce beliefs of “absolute obedience to one’s parents and to adults in general” or may impose that such conditions are caused by “spirit intrusion,” “violation of taboos,” “soul-loss,” or “disease sorcery.” It has also been documented that in certain communities and cultures, there is a strong reluctance to seek a genetic diagnosis since it may negatively impact their family’s prospects in terms of marriage, wealth, and/or wellbeing (Chediak et al., 2022).

The stigma associated with rare and genetic disorders further discourages patients to seek the needed support from their families or from healthcare professionals or genetic counselors and with the wider patient communities (Ayres et al., 2019).

## 5 Way forward

**FIGURE 2:**
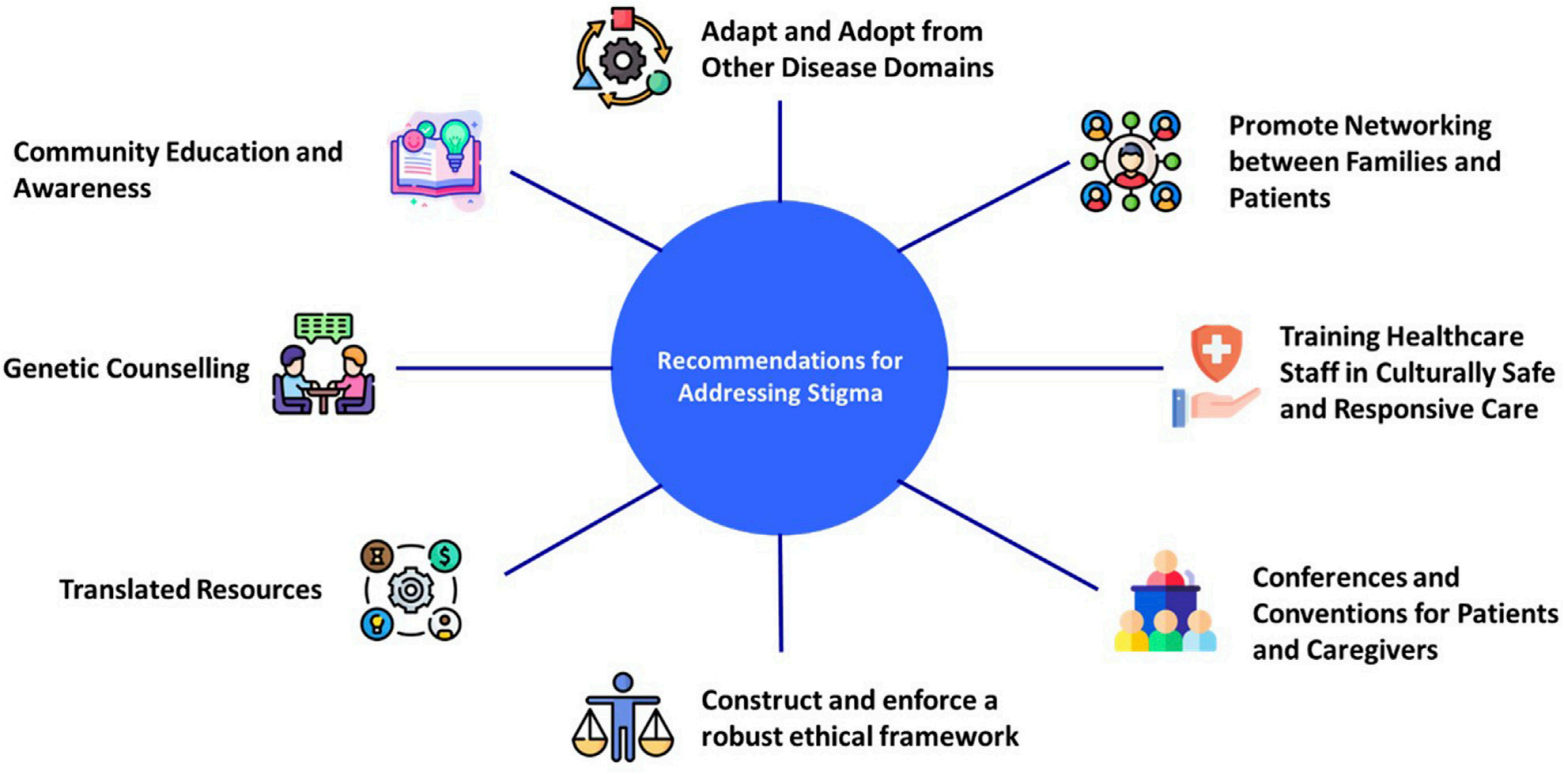
Recommendations for addressing stigma associated with genetic testing for RDs.

There are approximately 400 million people worldwide who are affected by RDs, and 71.9% of them have a genetic etiology. Hence, there is a palpable need for access to reliable education on rare and genetic diseases (Nguengang et al., 2020; Quinn et al., 2020). Furthermore, there is a need to incorporate emphasis on recognizing and addressing stigma in RD education. There is a crucial need to increase awareness among patients, carriers, families, and HCPs on RDs and genetic testing (NPHF, 2023). The authors believe that public education can be used to amend social norms, including reducing stigma. Public education needs to be disseminated in local languages, in addition to offering carrier screening in convenient (Xu et al., 2021) and culturally appropriate settings and with measures that proactively address the potential stigma. Often patients and their families are unable to find the necessary support to completely understand the diagnosis, implication of the results, and the management options available. The importance of appropriate pre- and post-genetic counseling, including addressing stigma, is extremely crucial for patients and their families. The importance of a planned clinical follow-up irrespective of the diagnostic outcome has been emphasized by the parents whose children have undergone genetic testing for RDs, and this provides a further opportunity for addressing stigma (Ayres et al., 2019). Figure 2 summarizes our recommendations for addressing stigma associated with genetic testing for RDs.

The authors believe that stigma impedes the research on social and behavioral factors associated with RDs exhibited by the patients and those around them. Awareness programs hence should be conducted for the general public, in addition to encouraging and empowering genetic counselors to provide their services to not only patients but also to their families and communities. This will help in addressing the inflamed stigmatization related to not only RDs but other genetic conditions. Genetic counselors, especially in countries with stronghold of taboos and superstitions, should be sensitized to the stigma and how appropriate interventions are crucial to overcoming it. The authors also concur that more work is needed in the future to develop a framework specific to RDs, which could entail a multistakeholder workshop to assess the full range of RD stakeholder’s views. Inputs from the existing framework along with clinical experience of the experts might help in addressing the gap in research on stigma associated with RDs and their genetic testing. A framework based on the published toolkit (National Institute of Mental Health, 2024), specific to RDs, will help set the benchmark for stigma assessment and tailor the approaches to address stigma as per the needs of the PLWRD.

As the scope of genetic testing is expanding for patients with RDs, LMICs have an opportunity to capitalize on these foundations and deliver greater equity and efficacy by taking a community-first approach that is tailored to the local context, including important cultural factors (Chediak et al., 2022). There is a need to empower primary healthcare providers through education to enable them to help PLWRDs, their caregivers, and families. Additionally, at a global scale, empowering nurses through education in counseling and stigma in addition to partnering nurses to genetic counselors can be considered through initiatives such as the global nursing network for RDs (NPHF, 2023). In order to support more people to make better informed choices about genetic testing and address concerns regarding privacy and discrimination, there is a need to construct and enforce a robust ethical framework. Genetic information should be de-identified, and privacy should be preserved in accordance with relevant jurisdictional practices, policies, and legislation. The laboratories and physicians handling genetic information should ensure public trust toward practices associated with data collection, storage, and appropriate data use. Additionally, there is a need to promote and enhance the dissemination of research findings to address apprehensions associated with genetic testing (Koromina et al., 2021). The process of data dissemination and more generally the underlying research should be co-designed with the population involved (D'Angelo et al., 2020). For conducting genetic counseling, similar to quality-of-life questionnaires, a survey to assess the stigma associated with genetic testing should be developed to identify and help address the unique counseling needs of PLWRDs and their families. A global taskforce to adapt and adopt from approaches to stigma in other domains and to ideate novel solutions could support personalized, as well as scalable approaches. Finally, a tailored toolkit similar to the Stigma and Discrimination Research toolkit, created by NIMH, can be developed, which addresses the nuances specific to RDs.

When addressing challenges experienced by PLWRDs associated with diagnosis and management, stigma related with genetic testing is not given a fair share of attention. The socioeconomic, educational, healthcare, and cultural differences lead to varied experiences for patients and families across the globe. Stigma associated with genetic testing adversely impacts timely diagnosis, receiving proper treatment/management, quality of life of patients and their parents/caregivers, and ultimately the patient outcomes. Deficient attention to stigma is an unmet need faced by many countries. It is hence imperative to address stigma linked with genetic testing to improve access to appropriate diagnostic tools and maximize health outcomes.

## References

[B1] Ando Y. Coelho T. Berk J. L. Cruz M. W. Ericzon B. G. Ikeda S. (2013). Guideline of transthyretin-related hereditary amyloidosis for clinicians. Orphanet J. Rare Dis. 8, 31. 10.1186/1750-1172-8-31 23425518 PMC3584981

[B2] Anton-Paduraru D.-T. (2017). NEWBORN SCREENING FOR CYSTIC FIBROSIS: TO SCREEN OR NOT TO SCREEN (PROS AND CONS). Breathe.

[B3] Ayres S. Gallacher L. Stark Z. Brett G. R. (2019). Genetic counseling in pediatric acute care: reflections on ultra-rapid genomic diagnoses in neonates. J. Genet. Couns. 28 (2), 273–282. 10.1002/jgc4.1086 30663825

[B4] Beck N. Applegate C. Ferreira C. (2020). Elements of genetic counseling for inborn errors of metabolism. Transl. Sci. Rare Dis. 4 (3-4), 197–208. 10.3233/trd-190044

[B5] Best S. Vidic N. An K. Collins F. White S. M. (2022). A systematic review of geographical inequities for accessing clinical genomic and genetic services for non-cancer related rare disease. Eur. J. Hum. Genet. 30 (6), 645–652. 10.1038/s41431-021-01022-5 35046503 PMC9177836

[B6] Boardman F. K. (2021). Attitudes toward population screening among people living with fragile X syndrome in the UK: 'I wouldn't wish him away, I'd just wish his fragile X syndrome away. J. Genet. Couns. 30 (1), 85–97. 10.1002/jgc4.1355 33184995 PMC7894324

[B7] Boardman F. K. Clark C. Jungkurth E. Young P. J. (2020). Social and cultural influences on genetic screening programme acceptability: a mixed-methods study of the views of adults, carriers, and family members living with thalassemia in the UK. J. Genet. Couns. 29 (6), 1026–1040. 10.1002/jgc4.1231 32114710 PMC7754126

[B8] Boardman F. K. Young P. J. Griffiths F. E. (2017). Population screening for spinal muscular atrophy: a mixed methods study of the views of affected families. Am. J. Med. Genet. A 173 (2), 421–434. 10.1002/ajmg.a.38031 27792846

[B9] Boardman F. K. Young P. J. Warren O. Griffiths F. E. (2018). The role of experiential knowledge within attitudes towards genetic carrier screening: a comparison of people with and without experience of spinal muscular atrophy. Health Expect. 21 (1), 201–211. 10.1111/hex.12602 28703871 PMC5750730

[B10] Boeldt D. L. Cheung C. Ariniello L. Darst B. F. Topol S. Schork N. J. (2017). Patient perspectives on whole-genome sequencing for undiagnosed diseases. Per Med. 14 (1), 17–25. 10.2217/pme-2016-0050 29749824

[B11] Boettcher J. Denecke J. Barkmann C. Wiegand-Grefe S. (2020). Quality of life and mental health in mothers and fathers caring for children and adolescents with rare diseases requiring long-term mechanical ventilation. Int. J. Environ. Res. Public Health 17 (23), 8975. 10.3390/ijerph17238975 33276595 PMC7731445

[B12] Bogart K. Hemmesch A. Barnes E. Blissenbach T. Beisang A. Engel P. (2022). Healthcare access, satisfaction, and health-related quality of life among children and adults with rare diseases. Orphanet J. Rare Dis. 17 (1), 196. 10.1186/s13023-022-02343-4 35549731 PMC9096775

[B13] Bonneau V. Nizon M. Latypova X. Gaultier A. Hoarau E. Bezieau S. (2021). First French study relative to preconception genetic testing: 1500 general population participants' opinion. Orphanet J. Rare Dis. 16 (1), 130. 10.1186/s13023-021-01754-z 33712027 PMC7955630

[B14] Boycott K. M. Ardigo D. (2018). Addressing challenges in the diagnosis and treatment of rare genetic diseases. Nat. Rev. Drug Discov. 17 (3), 151–152. 10.1038/nrd.2017.246 29242613

[B15] Catona D. Greene K. Magsamen-Conrad K. Carpenter A. (2016). Perceived and experienced stigma among people living with HIV: examining the role of prior stigmatization on reasons for and against future disclosures. J. Appl. Commun. Res. 44, 136–155. 10.1080/00909882.2016.1155726

[B16] Chediak L. Bedlington N. Gadson A. Kent A. Khalek A. A. Rosen L. (2022). Unlocking sociocultural and community factors for the global adoption of genomic medicine. Orphanet J. Rare Dis. 17 (1), 191. 10.1186/s13023-022-02328-3 35549752 PMC9097338

[B17] D’Angelo C. S. Hermes A. McMaster C. R. Prichep E. Richer E. van der Westhuizen F. H. (2020). Barriers and considerations for diagnosing rare diseases in indigenous populations. Front. Pediatr. 8, 579924. 10.3389/fped.2020.579924 33381478 PMC7767925

[B18] Dwyer A. A. Uveges M. K. Dockray S. Smith N. (2022). Exploring rare disease patient attitudes and beliefs regarding genetic testing: implications for person-centered care. J. Pers. Med. 12 (3), 477. 10.3390/jpm12030477 35330476 PMC8955005

[B19] Earnshaw V. A. Chaudoir S. R. (2009). From conceptualizing to measuring HIV stigma: a review of HIV stigma mechanism measures. AIDS Behav. 13 (6), 1160–1177. 10.1007/s10461-009-9593-3 19636699 PMC4511707

[B20] Earnshaw V. A. Smith L. R. Chaudoir S. R. Amico K. R. Copenhaver M. M. (2013). HIV stigma mechanisms and well-being among PLWH: a test of the HIV stigma framework. AIDS Behav. 17 (5), 1785–1795. 10.1007/s10461-013-0437-9 23456594 PMC3664141

[B21] Esquivel-Sada D. Nguyen M. T. (2018). Diagnosis of rare diseases under focus: impacts for Canadian patients. J. Community Genet. 9 (1), 37–50. 10.1007/s12687-017-0320-x 28733824 PMC5752651

[B22] Fagbemiro L. Adebamowo C. (2014). Knowledge and attitudes to personal genomics testing for complex diseases among Nigerians. BMC Med. Ethics 15, 34. 10.1186/1472-6939-15-34 24766930 PMC4005395

[B23] Ferreira C. R. (2019). The burden of rare diseases. Am. J. Med. Genet. A 179 (6), 885–892. 10.1002/ajmg.a.61124 30883013

[B24] Gallagher T. M. Bucciarelli M. Kavalukas S. L. Baker M. J. Saunders B. D. (2017). Attitudes toward genetic counseling and testing in patients with inherited endocrinopathies. Endocr. Pract. 23 (9), 1039–1044. 10.4158/EP171875.OR 28613942

[B25] Groft S. C. Posada M. Taruscio D. (2021). Progress, challenges and global approaches to rare diseases. Acta Paediatr. 110 (10), 2711–2716. 10.1111/apa.15974 34105798

[B26] IFPMA (2023). International federation of pharmaceutical manufacturers and associations. Available from: https://www.ifpma.org/subtopics/rare-diseases/#:∼:text=It%20is%20estimated%%0B20that%20one,and%20may%20be%20life%2Dthreatening (Accessed August 3, 2023). 10.1016/0895-4356(91)90112-m 2045841

[B27] James C. A. Hadley D. W. Holtzman N. A. Winkelstein J. A. (2006). How does the mode of inheritance of a genetic condition influence families? A study of guilt, blame, stigma, and understanding of inheritance and reproductive risks in families with X-linked and autosomal recessive diseases. Genet. Med. 8 (4), 234–242. 10.1097/01.gim.0000215177.28010.6e 16617244

[B28] Jun M. Thongpriwan V. Choi K. S. (2017). Experiences of prenatal genetic screening and diagnostic testing among pregnant Korean women of advanced maternal age. J. Transcult. Nurs. 28 (6), 550–557. 10.1177/1043659616662913 27510813

[B29] Kane J. C. Elafros M. A. Murray S. M. Mitchell E. M. H. Augustinavicius J. L. Causevic S. (2019). A scoping review of health-related stigma outcomes for high-burden diseases in low- and middle-income countries. BMC Med. 17 (1), 17. 10.1186/s12916-019-1250-8 30764819 PMC6376728

[B30] Kisanga E. Mutagonda R. Myemba D. T. Njiro B. J. Simon F. Marealle A. I. (2021). Premarital genetic screening and care of Tanzanian children with sickle cell disease: a qualitative study on parents' views and experiences. J. Community Genet. 12 (4), 515–523. 10.1007/s12687-021-00539-y 34287808 PMC8554897

[B31] Koromina M. Fanaras V. Baynam G. Mitropoulou C. Patrinos G. P. (2021). Ethics and equity in rare disease research and healthcare. Per Med. 18 (4), 407–416. 10.2217/pme-2020-0144 34085867

[B32] Kruse J. Mueller R. Aghdassi A. A. Lerch M. M. Salloch S. (2021). Genetic testing for rare diseases: a systematic review of ethical aspects. Front. Genet. 12, 701988. 10.3389/fgene.2021.701988 35154238 PMC8826556

[B33] Makarova E. V. Krysanov I. S. Valilyeva T. P. Vasiliev M. D. Zinchenko R. A. (2021). Evaluation of orphan diseases global burden. Eur. J. Transl. Myol. 31 (2), 9610. 10.4081/ejtm.2021.9610 33985324 PMC8274220

[B34] Munoz M. Sanz M. Perez-Santos E. Quiroga M. L. (2011). Proposal of a socio-cognitive-behavioral structural equation model of internalized stigma in people with severe and persistent mental illness. Psychiatry Res. 186 (2-3), 402–408. 10.1016/j.psychres.2010.06.019 20638731

[B35] Naik R. P. Haywood C. Jr (2015). Sickle cell trait diagnosis: clinical and social implications. Hematol. Am. Soc. Hematol. Educ. Program 2015 (1), 160–167. 10.1182/asheducation-2015.1.160 PMC4697437 26637716

[B36] National Institute of Mental Health (2024). Stigma and discrimination research toolkit: NIH: national Institute of mental health. Available from: https://www.nimh.nih.gov/about/organization/dar/stigma-and-discrimination-research-toolkit (Accessed February 27, 2024).

[B37] Nguengang W. S. Lambert D. M. Olry A. Rodwell C. Gueydan C. Lanneau V. (2020). Estimating cumulative point prevalence of rare diseases: analysis of the Orphanet database. Eur. J. Hum. Genet. 28 (2), 165–173. 10.1038/s41431-019-0508-0 31527858 PMC6974615

[B38] NPHF (2023). Connecting nurses across the World. Available from: https://gnnrd.org/ (Accessed August 10, 2023).

[B39] Phipps J. Skirton H. (2017). A qualitative study to explore the views and attitudes towards prenatal testing in adults who have Muenke syndrome and their partners. J. Genet. Couns. 26 (5), 1130–1142. 10.1007/s10897-017-0094-7 28332077

[B40] Primiero C. A. Yanes T. Finnane A. Soyer H. P. McInerney-Leo A. M. (2021). A systematic review on the impact of genetic testing for familial melanoma II: psychosocial outcomes and attitudes. Dermatology 237 (5), 816–826. 10.1159/000513576 33508831

[B41] Quinn L. Davis K. Yee A. Snyder H. (2020). Understanding genetic learning needs of people affected by rare disease. J. Genet. Couns. 29 (6), 1050–1058. 10.1002/jgc4.1233 32128950

[B42] RARE-X (2022). The power of being counted. Available from: https://rare-x.org/wp-content/uploads/2022/05/be-counted-052722-WEB.pdf (Accessed September 13, 2023).

[B43] Robillard J. M. Feng T. L. Kabacinska K. (2021). Access to genetic testing for rare diseases: existing gaps in public-facing information. World Med. Health Policy 13 (3), 518–525. 10.1002/wmh3.469 34692184 PMC8518969

[B44] Srinivasan S. Won N. Y. Dotson W. D. Wright S. T. Roberts M. C. (2020). Barriers and facilitators for cascade testing in genetic conditions: a systematic review. Eur. J. Hum. Genet. 28 (12), 1631–1644. 10.1038/s41431-020-00725-5 32948847 PMC7784694

[B45] Stangl A. L. Earnshaw V. A. Logie C. H. van Brakel W. L C. S. Barre I. (2019). The Health Stigma and Discrimination Framework: a global, crosscutting framework to inform research, intervention development, and policy on health-related stigmas. BMC Med. 17 (1), 31. 10.1186/s12916-019-1271-3 30764826 PMC6376797

[B46] Stoller J. K. (2018). The challenge of rare diseases. Chest 153 (6), 1309–1314. 10.1016/j.chest.2017.12.018 29325986

[B47] Tan R. K. J. Kaur N. Kumar P. A. Tay E. Leong A. Chen M. I. (2020). Clinics as spaces of costly disclosure: HIV/STI testing and anticipated stigma among gay, bisexual and queer men. Cult. Health Sex. 22 (3), 307–320. 10.1080/13691058.2019.1596313 30975036

[B48] The Global Genes (2023). Impact at a glance. Available from: https://globalgenes.org (Accessed September 13, 2023).

[B49] Tsai G. J. Cameron C. A. Czerwinski J. L. Mendez-Figueroa H. Peterson S. K. Noblin S. J. (2017). Attitudes towards prenatal genetic counseling, prenatal genetic testing, and termination of pregnancy among Southeast and East Asian women in the United States. J. Genet. Couns. 26 (5), 1041–1058. 10.1007/s10897-017-0084-9 28251433

[B50] U.S. Department of Health and Human Services (2023). Rare disease day at NIH 2023: NIH. Available from: https://ncats.nih.gov/news/events/rdd (Accessed September 12, 2023).

[B51] Verberne E. A. van den Heuvel L. M. Ponson-Wever M. de Vroomen M. Manshande M. E. Faries S. (2022). Genetic diagnosis for rare diseases in the Dutch Caribbean: a qualitative study on the experiences and associated needs of parents. Eur. J. Hum. Genet. 30 (5), 587–594. 10.1038/s41431-022-01039-4 35087185 PMC9091230

[B52] Walewski J. L. Donovan D. Nori M. (2019). How many zebras are there, and where are they hiding in medical literature? A literature review of publications on rare diseases. Expert Opin. Orphan Drugs 7 (11), 513–519. 10.1080/21678707.2019.1684260

[B53] Williams J. K. Erwin C. Juhl A. R. Mengeling M. Bombard Y. Hayden M. R. (2010). In their own words: reports of stigma and genetic discrimination by people at risk for Huntington disease in the International RESPOND-HD study. Am. J. Med. Genet. B Neuropsychiatr. Genet. 153B (6), 1150–1159. 10.1002/ajmg.b.31080 20468062 PMC3035936

[B54] Wojcik M. H. Bresnahan M. Del Rosario M. C. Ojeda M. M. Kritzer A. Fraiman Y. S. (2023). Rare diseases, common barriers: disparities in pediatric clinical genetics outcomes. Pediatr. Res. 93 (1), 110–117. 10.1038/s41390-022-02240-3 35963884 PMC9892172

[B55] World Health Organization (2019). Diabetes. Available from: https://www.who.int/news-room/fact-sheets/detail/diabetes (Accessed September 11, 2023).

[B56] Xu J. Z. Foe M. Tanongsaksakul W. Suksangpleng T. Ekwattanakit S. Riolueang S. (2021). Identification of optimal thalassemia screening strategies for migrant populations in Thailand using a qualitative approach. BMC Public Health 21 (1), 1796. 10.1186/s12889-021-11831-4 34615515 PMC8495975

[B57] Yabumoto M. Miller E. Rao A. Tabor H. K. Ormond K. E. Halley M. C. (2022). Perspectives of rare disease social media group participants on engaging with genetic counselors: mixed methods study. J. Med. Internet Res. 24 (12), e42084. 10.2196/42084 36542454 PMC9813816

[B58] Yau A. H. Y. Zayts O. A. (2014). ‘I don’t want to see my children suffer after birth’: the ‘risk of knowing’ talk and decision-making in prenatal screening for Down’s syndrome in Hong Kong. Health, Risk Soc. 16 (3), 259–276. 10.1080/13698575.2014.913008

[B59] Zanello G. Chan C. H. Pearce D. A. Group I. R. W. (2022). Recommendations from the IRDiRC Working Group on methodologies to assess the impact of diagnoses and therapies on rare disease patients. Orphanet J. Rare Dis. 17 (1), 181. 10.1186/s13023-022-02337-2 35526001 PMC9078009

[B60] Zelaya C. E. Sivaram S. Johnson S. C. Srikrishnan A. K. Suniti S. Celentano D. D. (2012). Measurement of self, experienced, and perceived HIV/AIDS stigma using parallel scales in Chennai, India. AIDS Care 24 (7), 846–855. 10.1080/09540121.2011.647674 22272891

[B61] Zhong A. Darren B. Loiseau B. He L. Q. B. Chang T. Hill J. (2021). Ethical, social, and cultural issues related to clinical genetic testing and counseling in low- and middle-income countries: a systematic review. Genet. Med. 23 (12), 2270–2280. 10.1038/s41436-018-0090-9 30072741

